# A Criterion-based Approach for the Systematic and Transparent Extrapolation of Clinical Trial Survival Data

**DOI:** 10.36469/9896

**Published:** 2015-02-14

**Authors:** Gabriel Tremblay, Patrick Haines, Andrew Briggs

**Affiliations:** 1 Eisai Co. Ltd., Woodcliff Lake, NJ, USA; 2 Curo Consulting, Marlow, Buckinghamshire, UK; 3 University of Glasgow, Glasgow, UK

**Keywords:** health technology assessment, metastatic breast cancer, criteria, piecewise models, parametric extrapolation, survival analysis

## Abstract

**Background:** Trial data often does not cover a sufficiently long period of time to truly capture time-toevent endpoints, however, Health Technology Assessment (HTA) bodies often require overall survival (OS) and progression-free survival (PFS) estimates. Often, significant survival effects are found beyond the time period observed in clinical trials, thus, extrapolation of trial results is required for health economic and HTA evaluations.

**Objectives:** This paper looks at different techniques that can be used to extrapolate trial data, as well as criteria that should be used to select the most appropriate technique. Using these insights a formal decisionmaking criteria will be established, allowing users to follow a systematic approach to extrapolating survival estimates. The techniques are then applied to a metastatic breast cancer (MBC) example.

**Methods:** A criterion-based guide was devised to allow the accurate extrapolation and justification of survival estimates in a MBC study comparing eribulin (Halaven) monotherapy with treatment of their (patient’s) physician’s choice (TPC). Parametric and piecewise models are used to extrapolate survival estimates, and statistical as well as visual tests are used to decide the most appropriate modelling technique.

**Results:** In the case study presented, the optimal model was identified as the Accelerated Failure Time (AFT) Parametric model using a Gamma distribution with a treatment covariate for OS, and the Kaplan-Meier survival estimates for PFS.

**Conclusions:** Survival estimates must be extrapolated to a time point such that the benefits of a therapy can be clearly demonstrated. A systematic approach combined with a formal decision-making structure should be used to minimize the potential for bias as well as making the process transparent.

## BACKGROUND

There is a growing need to extrapolate survival curves when the trial data prior to cut-off does not provide enough information on overall survival (OS) and progression free survival (PFS).[1] Health Technology Assessment (HTA) bodies require manufacturers of new health technologies to demonstrate the value of the product in terms of OS and PFS gain.[2] As a significant portion of the clinical value is reflected in the tail[2] of the survival curve, it is important to understand the different extrapolation techniques in order to ensure that the value of a new intervention can be appropriately presented.

The primary objective of this analysis is to compare extrapolation techniques by assessing the incremental difference between treatment arms. In addition, this analysis will determine the appropriate technique to be used in breast cancer to estimate OS and PFS for the dataset used. The mean incremental difference is used in this study because total cost or total survival can be computed from the mean[3], and because extreme values can be accounted for (unlike when using the median or other statistics).[4,5,6]

### Literature Review for Extrapolation of Survival in Economic mModels

Clinical trials can be limited in the data they provide as a result of time and/or budget constraints. Specifically, many trial results are published before the endpoint of interest (such as OS) is reached for all participants.[7] This can cause a "tip of the iceberg" effect where a significant proportion of the benefit is hidden after the trial end. In these cases, extrapolation of the available evidence is necessary to completely measure survival. Bias in the endpoint related to the effect of the assessment schedule is also a potential justification for extrapolation.[8] This bias is related to the data collection process and creates a stair-step look in the survival or PFS curves as the data is not collected continuously.

Traditionally, extrapolation involves the use of parametric models based on the regression analysis of patient-level data using either the exponential, Weibull, Log-normal, Log-logistic, Gamma or Gompertz distributions. Other model classes, such as piecewise models which are more flexible than traditional parametric models, are rarely used even if they can offer robust results in some cases.[1]

The difference between the two arms in the trial is often based on a treatment covariate. Some argue that both the shape and scale of the extrapolated function has to be modelled with precision, which suggests that a treatment covariate would not always be optimal.[9]

Different tests can be used to validate the optimal model class as well as the optimal model type or distribution within a class. These include visual examination of the hazard rate and the fit of the extrapolated survival.[10] The National Institute for Health and Care Excellence Decision Support Unit (NICE DSU) technical document explains that many studies do not present a solid case for their model choice. Another criticism is that the assumption of proportional hazards is often used without justification.[11] Also, uncertainty in the extrapolated estimates is often not modelled.[1]

### The Case Study

OS was the primary endpoint in a Phase 3 open-label randomized study of eribulin (Halaven) monotherapy versus a treatment of physician's choice (TPC) in patients with metastatic breast cancer (Eisai Metastatic Breast Cancer Study Assessing Physician's Choice versus Eribulin [EMBRACE] trial). In this study, women with locally recurrent or metastatic breast cancer were randomly given eribulin mesilate or TPC. The study is registered at ClinicalTrials.gov, #NCT00388726.[12] The study met its primary objective, showing a significant increase in OS for eribulin patients compared to TPC patients. Median overall survival was 13.1 months (11.8-14.3) in patients receiving eribulin and 10.6 months (95% confidence interval [CI] 9.3-12.5) in patients receiving TPC. These results also showed a significant increase in OS for eribulin compared to TPC patients, with a median OS of 13.2 months (95% CI 12.1-14.4) versus 10.5 months (9.2-12.0) with TPC.

## METHODOLOGY

### General Approach and Decision-making Criteria

The general approach described and used in this study was inspired by the NICE DSU Technical Report.[2] When there is an extrapolation need there are three steps to the approach, detailed in Appendix A.

The first step is the initial selection of the survival model before extrapolation has taken place. This tends to involve plotting the log-cumulative hazard of all treatment options and then using visual inspection to determine the appropriate model. The second step involves extrapolating survival results by using the appropriate model. In this stage, survival estimates are extrapolated to the end of the time horizon of interest for complete parametric models. Finally, the third step is to use statistical measures and decision criteria to select the model that most accurately models survival. Such criteria should not be seen as the answer to which model is most appropriate; instead they should be used to guide decisions.

### The Model Classes

In this analysis, three model classes are discussed. The first model class, the proportional hazards (PH) model, is relevant when the treatment effect is proportional over time. If this is true, the log-cumulative hazard plots of two (or more) arms will be parallel.[2] To model this hazard rate relation to time we include a treatment arm covariate which creates a "pattern" of hazard difference between each treatment, assuming that the hazard differential is constant over time. This is because the PH condition states that covariates are multiplicatively related to hazard. This class of model suggests a stable difference between each treatment arm.

In this first model class, we only considered (1) PH parametric models with a treatment covariate. Such survival modelling consists of fitting a distribution to the data, with the most common distributions in survival analysis including the Weibull, Exponential and Gompertz distributions.

Many economic analyses use a PH model to generate the marginal difference between treatment curves by applying a hazard ratio of treatment difference to the control arm of the trial.[11]

Model type (1) will be applied using three PH distributional forms; Weibull, Exponential and Gompertz. The Weibull distribution is monotonically increasing, decreasing or constant over time.

The next model class is recommended when log-cumulative hazard plots are not parallel, but relatively straight. For this model class this study focuses on (2) Accelerated Failure Time (AFT) models with a treatment covariate and (3) individual parametric models without a treatment covariate. Unlike PH models, AFT models assume that the effect of a covariate is to accelerate (or decelerate) the hazard by a constant, acting like a time-scaling factor.[13] Individual models are parametric models that do not use a treatment covariate, meaning the extrapolation for each arm is estimated separately. Individual models will also fit a single model type to the whole time frame of interest. Individual models are less flexible than piecewise models (discussed later), as the equation must fit the whole pre- and post-trial cut-off periods, but they avoid problems that can arise when transitioning between the pre- and post-trial cut-off.

With the previous models, survival estimates were extrapolated from the same analysis for both arms using a “treatment effect” covariate to generate the survival difference. With individual models each treatment arm will have survival results extrapolated on its own basis, allowing different distributions to be applied to each arm. Individual models can result in survival estimates for different treatment arms crossing. Sometimes, it is not clinically or statistically appropriate that this happens. However, because the shape and slope are determined by the pre cut-off trial data[2] the parametric extrapolation in the post cut-off period can lead to unrealistic cross-over. In other cases, the cross-over can be very important (for example, if cross-over occurs before the trial cut-off individual’s models should be used).

The final class discussed in this study includes piecewise and other flexible models. Such models are flexible enough to perform extrapolation when hazard rates are not constant over time, are monotonic or are non-monotonic.[2] Piecewise models are often amendments or hybrids, composed of one or more forms of survival modelling, including Kaplan-Meier survivor functions or parametric extrapolation. In 2011, only 2% of economic models used to estimate mean survival in NICE Technology Appraisals included piecewise survival extrapolation.[2] Piecewise modelling is recommended in the NICE DSU Technical Support Document on extrapolation when log-cumulative hazard plots lines are not straight. Non-straight lines mean that the hazard rate is time-dependent, and as such the proportional hazard assumption is not respected. This can be confirmed through the use of a global proportional hazard test.

Because piecewise modelling uses different extrapolation techniques at different time points, this kind of modelling could be less effective at extrapolating beyond the observed data.[2] In fact, piecewise survival mapping is often dynamic/non-constant or split into extrapolated sections separated by knots, which reduce its effectiveness at forecasting the survival with a proper tail, even if it can be effective at fitting the best curve to the observed data.[2] In this study, two techniques of this model class were examined. The first technique involves attaching parametric extrapolation survival estimates to the observed Kaplan-Meier curve.[14] The second technique used was the Royston & Parmar spline technique.[15]

The first model type tested in this class is (4) Kaplan-Meier survival function with an extrapolated tail. In this method, the observed data until trial cut-off (the first 34 months in the case study) are used to plot the Kaplan-Meier survival function. The first 34 months are therefore not extrapolated and the limitations of the Kaplan-Meier Survivor function remain valid. The data is then extrapolated past month 34, creating a ‘tail’ to the survival curve which is attached to the end of the Kaplan-Meier survival function. The tail is forecast using parametric models whilst assuming that the cut-off (in this example month 34) is in fact the first time period (month 0). Therefore, month 35 (cut-off + 1) is modelled assuming that it is month 1. To do this, the survivor function at month 34 is assumed to be at 100%. As an example, if 20% of the patients are still alive at month 34, and the parametric function indicates that the survival should be 100% at time 0 and 90% at time 1, the extrapolation of survival will be 18% (20% X 90%) at time “trial cut-off + 1”, or the 35th month. The extrapolated hazard rate could be constant or not, but also monotonic and could potentially lead to a change in hazard at the transition between the Kaplan-Meier curve and the extrapolated tail (between month 34 and 35). This puts emphasis on the marginal gain prior to extrapolation, which decreases the risk of exaggerating the extrapolated tail benefit.[2]

The second type of model tested within this class are (5) Royston and Parmar flexible models.[15] Royston and Parmar developed flexible parametric models, also called spline-based models, where the extrapolation is adjusted to have a different shape between a number of knots.[16] The number of knots, which define the boundary of the extrapolated section, can vary, but it is recommended to use six or less, creating seven areas. Adding knots impacts the degree of freedom and a large number of knots potentially adds more uncertainty than benefit.[16]

The classic method is to place the knots at predefined percentiles depending on the number of knots, with the distance between each knot being identical.[17] A second method would be to run 100 random knot placements and select the placements that give the lowest deviance (best Akaike information criterion [AIC], Bayesian information criterion [BIC]). As the selection is random, there is no guarantee that the random placement selected is the "best" one, so we decided to use a Durrleman & Simon approach (the classic method). To select the optimal number of knots, an AIC/BIC comparison was used as in the Royston paper.[16] Two types of models can be used within the Royston & Parmar framework; the PH model using a Weibull distribution and the proportional odd (PO) model, using a log-logistic distribution.[15]

## Decision Criteria

This section establishes decision criteria for selecting the optimal model for use in extrapolating outcomes.

First, analyzing the data using statistical tests and visual inspection is necessary to understand the dataset and help guide modelers toward the best-fitting model. These occur before extrapolation has taken place.

**Criterion 1 – Proportional Hazard Assumption Testing:** The PH assumption has to be strongly supported by the log-cumulative hazard plots and the PH global statistics if the selected model advocates this assumption.[11,2,18]

Most published work on survival extrapolation does not use the log-cumulative hazard plots to evaluate the PH assumption[2], even though it is an important decision-making criteria for the best-fitting model[11] and is recommended in the NICE DSU technical report. First, if the log-cumulative hazard line is not straight then the hazard is not constant. Secondly, if the log-cumulative hazard plots of two treatment arms are parallel, then the hazard rate in both arms have a similar relationship to time. These two tests help to identify which class of model is preferable. The log cumulative hazard lines of the arms may cross, suggesting that one arm has an acceleration of the hazard rate at some point and giving a converging (or diverging) effect on the survival functions. In this case the use of a treatment effect covariate, which assumes a stable relative difference, will cause an extrapolation bias.

This study added a systematic process to assess the PH assumption, using a statistical test in addition to visual examination. We used the PH global test, which can be used to evaluate the goodness of fit of the data using the Schoenfeld residuals.[18] This method can be considered more objective than a plot comparison by visual inspection, which does not follow a systematic approach.[18] A significant result for this test indicates a deviation from the PH assumption.

Once the tests of criterion 1 are performed, the results of the extrapolation can be compared, and criteria 2 to 5 allow us to select the best model.

**Criterion 2 – Extrapolated Hazard Function fitting in Time and between Trial Arms:** The hazard rates have a similar time relation pattern between the extrapolation function and the Kaplan-Meier survivor function. The characteristic of the relation between the hazard rate of both arms are replicated by the modelling technique selected e.g. crossing lines would suggest an individual parametric model.

Visual examination is one of the most common "fitting" comparison methods in survival extrapolation.[2] Two types of visual examination should be performed to ensure proper fitting. First, the fitting of the extrapolated hazard curve to the Kaplan-Meier hazard curve must be examined. This allows the modeler to see if a characteristic of the curve is not represented in the distributional form used, and guides towards the most accurate model if so. The second method of visual inspection involves identifying if the relationship between the extrapolated hazard function within the treatment arms fit well with the hazard relationship examined in criterion 1. Models where the hazard patterns seem to fit should be prioritized.

**Criterion 3 – Minimal AIC and BIC:** For parametric models, the selected model must have a low AIC/BIC to demonstrate its goodness-of-fit to the survival curve in the pre-extrapolation period.

AIC and BIC are fitting statistics that are often used to compare parametric models. They are a measure of the relative quality of the statistical model, but models are rarely exclusively selected based on this method.[11] Nevertheless, this technique represents an effective way to evaluate the general fitting of the model based on the data available, but should be used in combination with other selection criteria. The tests from criterion 1 to 3 have one major flaw: As these tests focus on the comparison of the pre-extrapolation data, they do not test the post-extrapolation period.[2] The post-extrapolation period could affect the estimated survival results and cause significant bias. Therefore, other comparison methods are needed. In this analysis, we included two additional methods, which are described in criterion 4 and 5.

**Criterion 4 – Uncertainty in the Results:** Uncertainty should be accounted for when selecting the best model, as a high uncertainty would be a sign of low robustness.

To assess uncertainty, the CIs surrounding the marginal difference in survival between the treatment arms are used. Uncertainty can be measured using a bootstrap method to evaluate the CIs of the estimates.[8]

The nature of the different extrapolation techniques can have an impact on the estimates. Nevertheless, experts should be careful in selecting models with wide CIs.

**Criterion 5 – Similitude of Pre-extrapolation Marginal Gain and Realism of the Extrapolated Marginal Gain:** The realism of the marginal gain should be accounted for when selecting the best model as an unrealistic marginal gain would create bias in the economic analysis.

Criterion 5 is the simple comparison of the pre and post extrapolation area under the curve. In the best-case scenario, the pre-extrapolation result has a strong AIC/BIC fit, and for the post-extrapolation period the marginal gain should not overestimate the difference between the curves. An inflated difference between the two arms caused by an erroneous extrapolation could create a bias in the analysis.

To evaluate the realism of the post-extrapolation survival gain, we used a 'rule-of-thumb', stating that the ratio of the marginal relative difference in the extrapolated period (post cut-off) divided by the number of months post-cut-off should not be higher than the ratio of marginal difference on the number of months in the pre-extrapolation period. In other words, the average "rate of survival gain" per month between treatments should be equal or inferior in the post-extrapolation period compared to the pre-extrapolation period. This simple calculation provides a maximum realistic gap between the two arms, but it should only be used as a rule-of-thumb. As an example, if the marginal difference in the pre-extrapolation period is two months over a 34 months period, the "rate of survival gain" is 0.0588 of marginal gain per month. If we assume that the result of the extrapolation over 60 months is 3 months difference between treatments, then 1 month gain is generated over the last 26 months, which gives a "rate of survival gain" of 0.0384. The rate is smaller than the pre-extrapolation "rate of survival gain," and therefore satisfies the rule of thumb. Another characteristic that should be looked at is the realism of the total marginal gain. If the total marginal gain is lower than the pre-extrapolation marginal gain, it should be explained in detail using the hazard trend or by showing a crossing point in the survival data.

## METASTATIC BREAST CANCER CASE STUDY

At the last trial observation, 22.65% of patients in the eribulin arm remained alive, compared to 18.47% for the TPC arm. The primary reason for justifying extrapolation is a lack of trial data for a sufficiently long period that is able to highlight marginal gains in overall survival. Because the uncertainty in survival increases as we move to the right of a Kaplan-Meier curve, it is sometimes suggested that the last observations be removed as they can significantly affect the results of extrapolation, however the NICE DSU does not recommend this.[2] In this case study, the data analysis stopped at the trial cut-off, resulting in the exclusion of one patient. The survival curve quickly loses its precision after the trial cut-off date and sharply falls to 4.49% when the last uncensored eribulin patient dies, even if 22.65% of eribulin patients were still alive at the end of the trial. The actual difference between the survival rates in the trial was at 4.2% in favor of eribulin, and the survival results at the trial cut-off was respectively at 13.5% and 6.4% for eribulin and TPC, which seemed more realistic for the extrapolation. As the curves are not crossing in the actual data, such crossing generated by one patient would generate a bias when extrapolating using the piecewise models attached at the tail of the Kaplan-Meier survivor function (See Appendix B).

With regard to PFS, all patients had reached progression at month 16 in the eribulin group and at month 18 in the TPC group. As such, there is no need for extrapolation post-cut-off in this case as each individual reached the endpoint within the observed trial period. In this case, the Kaplan-Meier survival function proved to be the optimal function to measure PFS in an economic analysis (see Appendix B).

The log-cumulative hazard plots were then analyzed to detect the hazard patterns and identify the optimal model class (Figure 1). For OS, the lines are relatively straight, and relatively parallel. However, the hazard plots for both treatment arms cross, diverge, and then converge again, meaning the validity of all models should be checked using the other decision making criteria. The global proportional hazard test based on residuals does not show a significant result (p-value=0.0845), which indicates that the curves do not deviate from the PH assumption, but the p-value is far from convincing enough to justify using the PH assumption with no other justification.

**Figure 1.** Log-cumulative Hazard Plots of Overall Survival and Progression-free Survival of Eribulin Mesylate and TPC

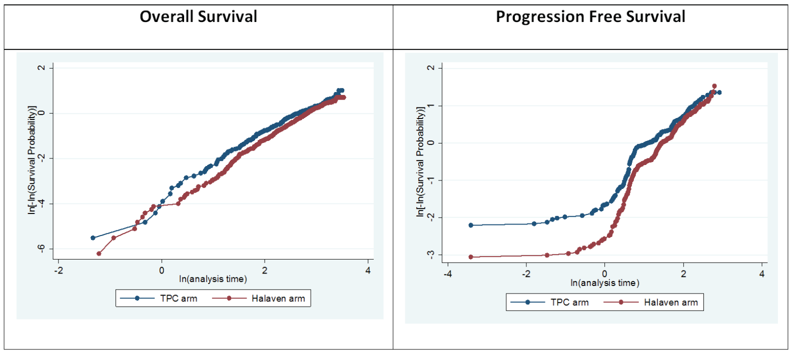

TPC: treatment of physicians choice

## Comparison of Approach

Table 1 presents the OS results for all the extrapolation techniques examined in our general approach, along with statistics related to our criterion based approach.

## Selection based on Decision Criteria

**Criterion 1 – PH Assumption Testing:** The PH assumption appears to be a fair assumption for overall survival, as tested by visual inspection and the global statistics. Regardless, the crossing of both lines and an apparent convergence could affect the hazard results.

As the PH assumption seems appropriate, the PH and accelerated failure time models can be used. As the visual analysis highlighted some potential problems with hazard rate plots, it is important to also consider the other model classes and types. The PH model with treatment covariates and AFT/individual models were the optimal model class. The next step is to evaluate the modeling techniques by comparing extrapolation results:

When plots are parallel:

1) **PH Parametric Model with Treatment Covariate:** The Weibull distribution gives similar hazard patterns to the Kaplan-Meier function. In this analysis, a treatment covariate was included, which ensures a rational relationship between the hazards of each arm (Criteria 2). The AIC/BIC evaluation indicated that the Weibull distribution is the best fit (Criterion 3). The level of uncertainty is quite high, but the lower CI does not cross 0 (Criterion 4). Criterion 5 was tested using two "rates of survival gain" to compare to the post-extrapolation rate. The first one was the Kaplan-Meier rate, which was of 0.0581 per month, and the second was the pre-extrapolation "rate of survival gain" of the extrapolation results. Criterion 5 was satisfied in both cases with an average of 0.016 month (Criterion 5). Furthermore, the post-extrapolation period respects the thumb rule and seems conservative (Criterion 5). Results suggest that the Weibull distribution is the best fit for the PH models.

When plots are not parallel:

2) **Accelerated Failure Time with Treatment Covariates:** Both the Log-logistic distribution and the Gamma distribution have similar hazard patterns to that of the Kaplan-Meier function and are based on a treatment covariate model, which ensures a rational relationship between the hazard plots of each arm (Criteria 2) (Figure 2 presents an example of the log-cumulative hazard plots for Halaven of the extrapolated function to evaluate criterion 2 for the complete parametric models). The level of uncertainty is lower than in the PH models (Criterion 4), and the log-logistic and Gamma distributions have the best AIC/BIC profile (Criterion 3). The extrapolation realism thumb rule was satisfied with an average of 0.026 month (Criterion 5). The post extrapolation period respects the thumb rule and seems conservative (Criterion 5). The results suggest that the best model here uses the Gamma distribution followed by the Log-Logistic, but the Gamma has a more conservative marginal gain, and long tails are not to be expected for metastatic breast cancer - as such, we would recommend the Gamma distribution.

**Table 1.** Overall Survival Extrapolation Results

| Model Type | Techniques | Sub-technique | Area between the Curves: OS Pre Cut-off | Area between the Curves: OS Post Cut-off Extrapolated Tale | Area between the Curves: Total Difference in OS | Fitting Statistics: Lower Bound CI | Fitting Statistics: Higher Bound CI | Fitting Statistics: Fitting Statistics (AIC/BIC) |
|---|---|---|---|---|---|---|---|---|
| **Plots are Parallel** | **(1) PH Parametric models with treatment covariate** | Weibull | 1.66 | 0.36 | 2.03 | 0.14 | 3.98 | 1947 / 1961 |
| | | Exponential | 1.70 | 0.71 | 2.41 | 0.33 | 5.11 | 2008 / 2017 |
| | | Gompertz | 1.63 | 0.24 | 1.87 | 0.10 | 3.53 | 1980 / 1994 |
| **Plots are not Parallel** | **(2) AFT Parametric models with treatment covariates** | Log-Normal | 2.22 | 0.80 | 3.02 | 1.26 | 6.57 | 1953 / 1967 |
| | | Log-Logistic | 2.20 | 0.68 | 2.88 | 1.34 | 7.11 | 1936 / 1950 |
| | | Gamma | 2.00 | 0.58 | 2.57 | 0.57 | 4.97 | 1936 / 1955 |
| | **(3) Individual parametric models** | Weibull | 1.76 | 0.07 | 1.83 | -0.26 | 3.93 | 1946 / 1962 |
| | | Log-Normal | 1.89 | 0.39 | 2.28 | -1.41 | 5.96 | 1954 / 1969 |
| | | Log-Logistic | 1.88 | 0.22 | 2.10 | -2.82 | 7.03 | 1936 / 1952 |
| | | Exponential | 1.70 | 0.71 | 2.41 | -0.67 | 5.48 | 2008 / 2016 |
| | | Gamma | 1.79 | -0.11 | 1.68 | -2.13 | 5.49 | 1937 / 1960 |
| | | Gompertz | 2.62 | -0.05 | 2.57 | 0.68 | 4.47 | 1980 / 1996 |
| **Plots are not Straight Lines: Consider Piecewise Models** | **(4) SF + parametric tale extrapolation** | Weibull | 1.98 | 1.06 | 3.04 | 2.74 | 3.39 | 1947/ 1961* |
| | | Log-Normal | 1.98 | 1.09 | 3.07 | 2.86 | 3.32 | 1953/ 1967* |
| | | Log-Logistic | 1.98 | 1.09 | 3.07 | 2.92 | 3.39 | 1936/ 1950* |
| | | Exponential | 1.98 | 1.02 | 3.00 | 2.75 | 3.29 | 2008/ 2017* |
| | | Gamma | 1.98 | 1.08 | 3.05 | 2.77 | 3.39 | 1936/ 1955* |
| | | Gompertz | 1.98 | 1.05 | 3.03 | 2.74 | 3.36 | 1980/ 1994* |
| | **(5) Royston & Parmar flexible models** | Weibull PH with 1 node | 1.78 | -0.07 | 1.72 | 0.15 | 3.52 | 0 / 0 |
| | | Log-Logistic PO with 1 node | 1.77 | -0.02 | 1.75 | 0.17 | 3.55 | 0 / 0 |

OS: overall survivial; CI: confidence interval; AIC: Akaike information criterion; BIC: Bayesian information criterion; PH: proportional hazard; AFT: accelerated failure time; SF: survival function; PO: proportional odd (model)

3) **Complete Parametric Extrapolation of the Individual Model:** The Log-logistic, Gamma and Weibull distribution have similar hazard patterns to the Kaplan-Meier estimator (Criteria 2). A converging hazard rate is realistic given the data, but crossing of the survival estimates is present for some of the distributions, which is not reflected in the data and would create a bias in the results (criterion 5). The level of uncertainty appears very high, with the lower bound of the CI crossing 0 for all extrapolations (Criteria 4). The AIC/BIC evaluation indicated that the Log-logistic and Gamma distributions provide the best fit (Criterion 3). The extrapolation realism thumb rule was satisfied with an average of 0.007 month (Criterion 5). The post-extrapolation period respects the thumb rule and seems very conservative, except for the Gamma distribution, which is negative. This negative effect can be caused by the convergence of the hazard rate at the end of the pre-extrapolation period, but the survival curve should cross post-extrapolation (Criterion 5). Therefore, the Log-logistic distribution should be used for this model type.

**Figure 2.** Log-cumulative Plots of the Extrapolated Function for Halaven for the Complete Parametric Models

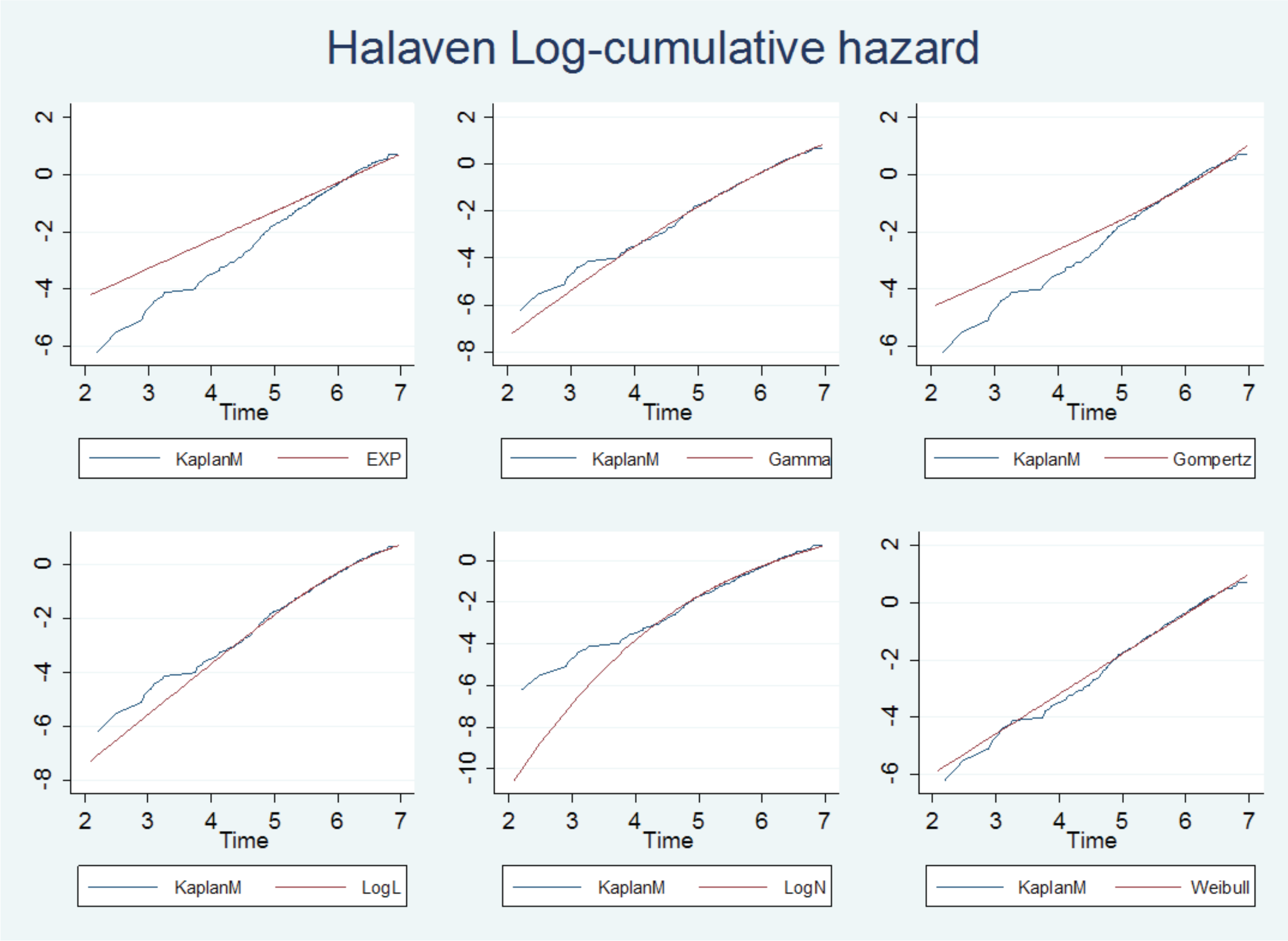

When Plots are not straight lines:

4) **Survival Function + Parametric Tail Extrapolation:** Log-logistic, Gamma, and also Weibull distributions have similar hazard patterns to the Kaplan-Meier estimator and are based on a treatment covariate model, which ensure a rational relationship between the hazard of each arms (Criteria 2). The AIC/BIC indicate that the Log-logistic, Gamma and Weibull distributions provide the best fit (Criterion 3). Assuming no uncertainty in the Kaplan-Meier portion of the curve, the level of uncertainty in the extrapolated section is limited (Criterion 4). The thumb rule was satisfied with an average of 0.040 month (Criterion 5). All extrapolations are close to the thumb rule threshold (Criterion 5). The Gamma distribution displays the best fitting profile in this extrapolation class.

5) **Royston and Parmar Flexible Models:** The PO and PH models offer very similar results and hazard patterns to the Kaplan-Meier function (Criterion 2). Both models have a good AIC/BIC profile (Criterion 3). The level of uncertainty is similar to the PH models with covariates, and the CIs do not cross 0 (Criteria 4). The extrapolation realism thumb rule was satisfied with an average of -0.001 month (Criterion 5). Both models project a negative post-extrapolation difference, which is not suggested by the data (Criterion 5). Due to the crossing problem, we would not recommend using these extrapolation techniques in this context. The results show that for a PH model, one knot should be used (lowest AIC and BIC), results are displayed in Appendix C. For the PO model, no knots should be used, which is equivalent to using an individual parametric model.

### Selecting the Optional Model for Overall Survival

In the optimal model class (the PH model class where plots are parallel), the Weibull model with use of a treatment covariate is the superior model. In the individual and AFT model class (where plots are not parallel), the Gamma AFT model with use of a treatment covariate is superior. In the Piecewise model class (plots are not straight lines), the Gamma tail attached to the survival function is the superior model. Among all classes, we would recommend the use of a Gamma AFT model with a treatment covariate because it has a better AIC/BIC profile, less uncertainty and a strong realism of the pre- and post-extrapolation area under the curve. Appendix D presents the model comparison based on our criteria.

## DISCUSSION AND CONCLUSION

The comparison of the different classes of models allows us to discuss the limitations and advantages of each type of model. The case study used enables us to highlight some concerns about different types of methodology, but similar studies should be performed using this technique.

Below are some key pieces of information discussed through this study:

- Kaplan-Meier survivor functions can be widely affected by the last observation, and do not offer any form of extrapolation beyond the last observation.
- Visual examination is important, but statistical tests and technical decision criteria are needed.
- PH parametric models are inherently coherent with the need to evaluate a difference between the arms survival curves. Validating the PH assumption is critical to the use of these models.
- Individual and AFT models can offer a good alternative to PH models when the PH assumption is not validated.
- More flexible models, such as piecewise models, can offer a robust alternative to PH/AFT or individual models when hazard rates do not follow clear patterns.
- Analysts looking to extrapolate survival data should compare models following a clear process, and not restrict themselves to simpler models.

In the case study presented, the optimal model was identified as the AFT Parametric model using a Gamma distribution with a treatment covariate for overall survival, and the Kaplan-Meier estimator for PFS.

Having a strong decision-making process is important and can avoid generating bias. Selecting a method that is conservative and in line with the criteria should be a priority, rather than presenting the method that provided the highest marginal benefit.

Finally, the optimal model should be included in economic analysis or HTA analysis under the base case scenario, but the second best model should be included in the sensitivity analysis.

### Conflict of Interest Declaration

The study was supported by Eisai Ltd. GT is an employee of Eisai Ltd. PH and AB received funding from Eisai for their contribution to the study.

## APPENDICES

### Appendix A

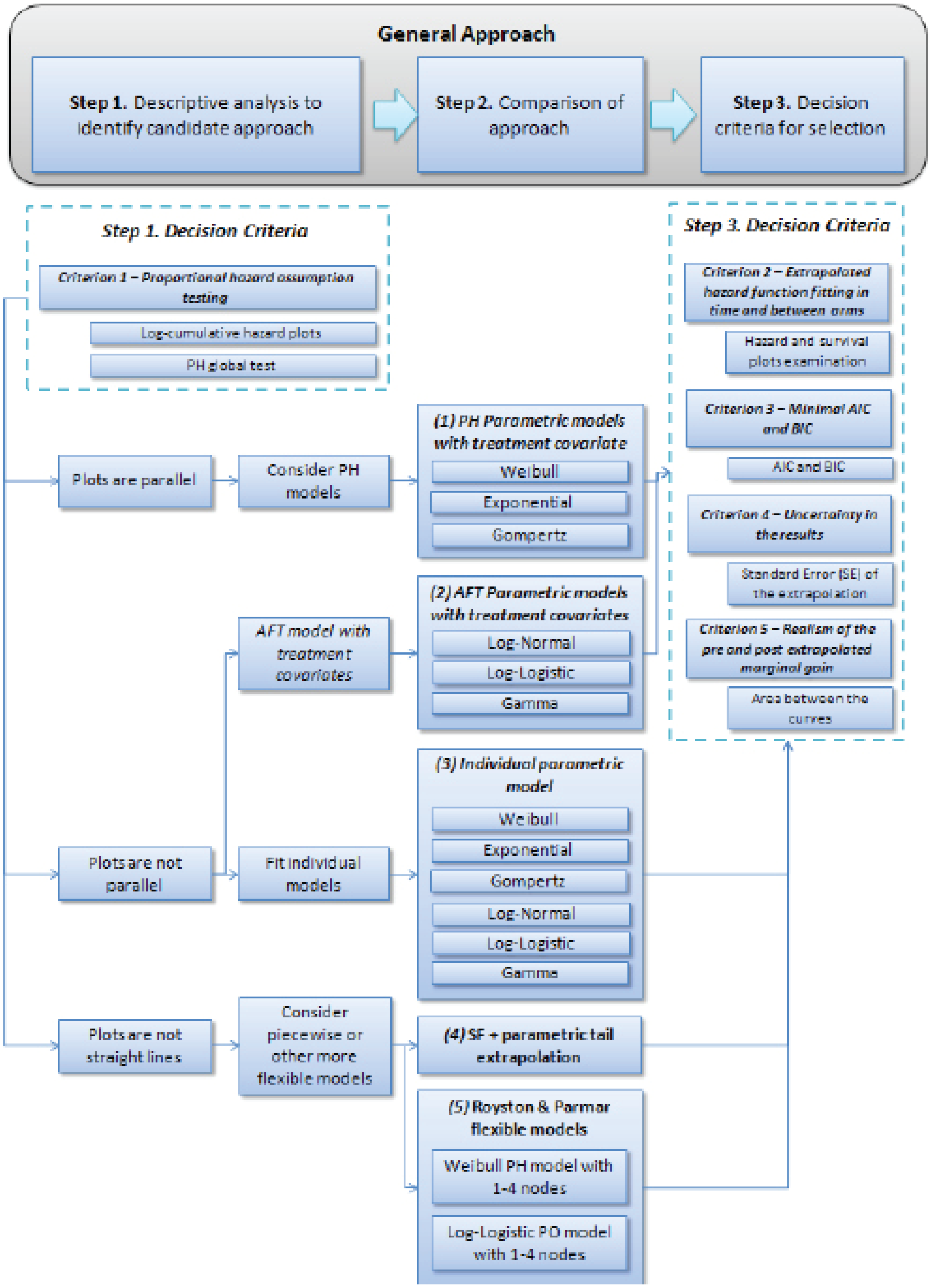

PH: proportional hazard; AFT: accelerated failure time; SF: survival function

## Appendix B

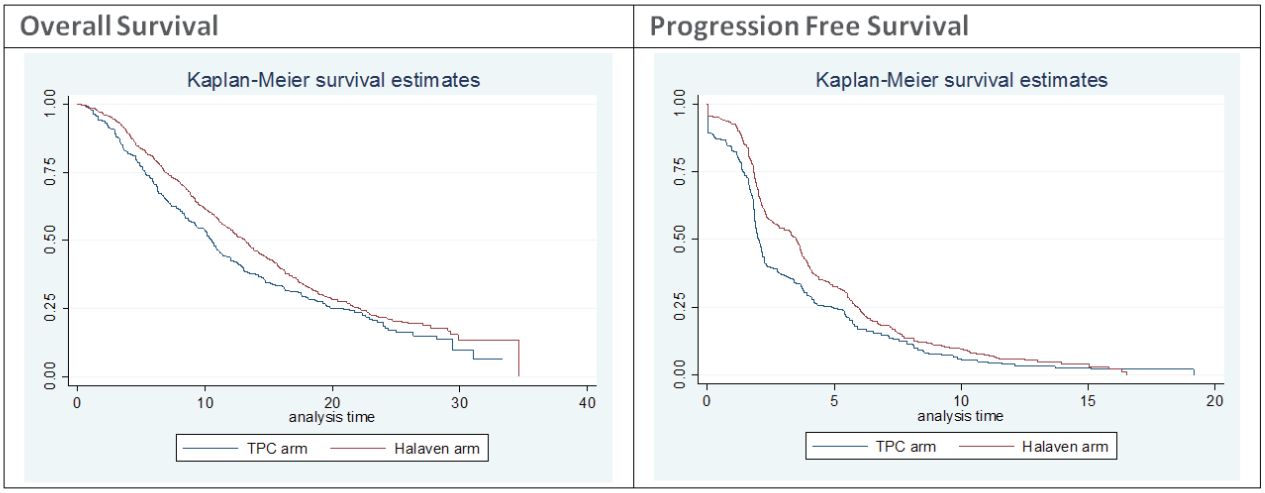

## Appendix C

| | Proportional Hazard | | Proportional Odd | |
|---|---|---|---|---|
| **Knots** | **AIC** | **BIC** | **AIC** | **BIC** |
| 0 | 1946.4 | 1961.9 | 1936.4 | 1951.8 |
| 1 | 1937.2 | 1960.4 | 1938.2 | 1961.4 |
| 2 | 1940.2 | 1971.1 | 1940.8 | 1971.7 |
| 3 | 1943.8 | 1982.4 | 1944.8 | 1983.5 |
| 4 | 1946.9 | 1993.3 | 1948.2 | 1994.6 |

AIC: Akaike information criterion; BIC: Bayesian information criterion

## Appendix D

| | Criteria | | | | | Recommendation |
|---|---|---|---|---|---|---|
| **Model Types** | **1** | **2** | **3** | **4** | **5** | |
| (1) PH Parametric models with treatment covariate | √ | √ | √ | √ | ! | Weibull |
| (2) AFT Parametric models with treatment covariates | √ | √ | √ | √ | ! | Gamma |
| (3) AFT individual parametric model | √ | ! | √ | ! | ! | Log-logistic |
| (4) SF + parametric tale extrapolation | **NA** | √ | √ | √ | √ | Gamma |
| (5) Royston & Parmar flexible models | √ | √ | √ | √ | | 1 knot |

PH: proportional hazard; AFT: accelerated failure time; SF: survival function

## References

[1] Jackson CH, Sharples LD, Thompson SG: Survival models in health economic evaluations: Balancing fit and parsimony to improve prediction. *Int J Biostat.* 2010;6(1):Article 34.

[2] Latimer N: Survival analysis for economic evaluations alongside clinical trials - Extrapolation with patient-level data. NICE DSU Technical Support Document 14. Sheffield: Decision Support Unit, ScHARR, University of Sheffield, 2013.

[3] Zhou XH, Melfi CA, Hui SL: Methods for comparison of cost data. *Ann Intern Med.* 1997;752–6.

[4] Bang H, Zhao H: Median-based incremental cost-effectiveness ratio (ICER). *J Stat Theory Pract.* 2012;6(3):428–42.

[5] Siegel JE, Weinstein MC, Russell LB, Gold MR: Recommendations for reporting cost-effectiveness analyses. Panel on cost-effectiveness in health and Medicine. *JAMA.* 1996;276:1339–41.

[6] Guyot P, Ades AE, Ouwens MJ, Welton NJ: Enhanced secondary analysis of survival data: reconstructing the data from published Kaplan-Meier survival curves. *BMC Med Res Methodol.* 2012;12:9.

[7] Annemans L: Outcomes Assessment: Extrapolation in Oncology Modelling: Novel Methods for Novel Compounds. *ISPOR Connections.* August 2012. http://www.ispor.org/news/articles/Aug12/Extrapolation-in-Oncology-Modelling.asp. Accessed September 7, 2014.

[8] Panageas KS, Ben-Porat L, Dickler MN, Chapman PB, Schrag D: When you look matters: The effect of assessment schedule on progression-free survival. *J Natl Cancer Inst.* 2007;99(6):428-32.

[9] Ouwens MJ, Philips Z, Jansen JP: Network meta-analysis of parametric survival curves. *Research Synthesis Methods.* 2010;1:258-71.

[10] Royston P, Parmar MKB, Altman DG: External validation and updating of a prognostic survival model. Hub for Trials Methodology Research, MRC Clinical Trials Unit and University College London, 17 March 2010. https://www.ucl.ac.uk/statistics/research/pdfs/rr307.pdf.

[11] Guyot P, Welton NJ, Ouwens MJ, Ades AE: Survival time outcomes in randomized, controlled trials and meta-analyses: The parallel universes of efficacy and cost-effectiveness. *Value Health.* 2011;14(5):640-6.

[12] Cortes J, O'Shaughnessy J, Loesch D, et al: Eribulin monotherapy versus treatment of physician's choice in patients with metastatic breast cancer (EMBRACE): A phase 3 open-label randomised study. *Lancet.* 2011;377(9769):914–23.

[13] Jenkins SP: Survival Analysis with Stata. Colchester: Institute for Social and Economic Research (ISER), University of Essex, 2008. https://www.iser.essex.ac.uk/resources/survival-analysis-with-stata.

[14] Billingham LJ, Abrams KR, Jones DR. Methods for the analysis of quality-of-life and survival data in health technology assessment. *Health Technol Assess.* 1999;3(10):1-152.

[15] Royston P, Parmar MK: Flexible parametric proportional-hazards and proportional-odds models for censored survival data, with appication to prognostic modelling and estimation of treatment effects. *Stat Med.* 2002;21(15):2175–97.

[16] Royston P: Flexible parametric alternatives to the Cox model, and more. *Stata Journal.* 2001;1(1):1–28.

[17] Durrleman S, Simon R: Flexible regression models with cubic splines. *Stat Med.* 1989;8(5):551-61.

[18] Abeysekera WWM, Sooriyarachchi MR: Use of Schoenfeld's global test to test the proportional hazards assumption in the cox proportion hazards model: an application to a clinical study. *J Natn Sci Foundation Sri Lanka.* 2009;37(1):41-51.
